# Efavirenz reduces renal excretion of lamivudine in rats by inhibiting organic cation transporters (OCT, Oct) and multidrug and toxin extrusion proteins (MATE, Mate)

**DOI:** 10.1371/journal.pone.0202706

**Published:** 2018-08-16

**Authors:** Martina Ceckova, Josef Reznicek, Birgit Deutsch, Martin F. Fromm, Frantisek Staud

**Affiliations:** 1 Department of Pharmacology and Toxicology, Charles University in Prague, Faculty of Pharmacy in Hradec Kralove, Hradec Kralove, Czech Republic; 2 Institute of Experimental and Clinical Pharmacology and Toxicology, Friedrich-Alexander-Universität Erlangen-Nürnberg, Erlangen, Germany; University of Kentucky, UNITED STATES

## Abstract

Efavirenz (EFV) is a non-nucleoside reverse transcriptase inhibitor used in first-line combination antiretroviral therapy (cART). It is usually administered with nucleoside reverse transcriptase inhibitors (NRTI), many of which are substrates of OCT uptake solute carriers (SLC22A) and MATE (SLC47A), P-gp (MDR1, ABCB1), BCRP (ABCG2), or MRP2 (ABCC2) efflux transporters. The aim of this study was to evaluate the inhibitory potential of efavirenz towards these transporters and investigate its effects on the pharmacokinetics and tissue distribution of a known Oct/Mate substrate, lamivudine, in rats. Accumulation and transport assays showed that efavirenz inhibits the uptake of metformin by OCT1-, OCT2- and MATE1-expressing MDCK cells and reduces transcellular transport of lamivudine across OCT1/OCT2- and MATE1-expressing MDCK monolayers. Only negligible inhibition of MATE2-K was observed in HEK-MATE2-K cells. Efavirenz also reduced the efflux of calcein from MDCK-MRP2 cells, but had a rather weak inhibitory effect on Hoechst 33342 accumulation in MDCK-MDR1 and MDCK-BCRP cells. An *in vivo* pharmacokinetic interaction study in male Wistar rats revealed that intravenous injection of efavirenz or the control Oct/Mate inhibitor cimetidine significantly reduced the recovery of lamivudine in urine and greatly increased lamivudine retention in the renal tissue. Co-administration with efavirenz or cimetidine also increased the AUC_0-∞_ value and reduced total body clearance of lamivudine. These data suggest that efavirenz is a potent inhibitor of OCT/Oct and MATE/Mate transporters. Consequently, it can engage in drug-drug interactions that reduce renal excretion of co-administered substrates and enhance their retention in the kidneys, potentially compromising therapeutic safety.

## Introduction

Efavirenz is one of the most widely used non-nucleoside reverse transcriptase inhibitors (NNRTI) in the treatment of human immunodeficiency virus 1 (HIV-1)-infected adults and children [1]. Co-administration of efavirenz with nucleoside reverse transcriptase inhibitors (NRTI), namely tenofovir disoproxil fumarate and lamivudine, or alternatively, emtricitabine, is currently the preferred first-line regimen of combination antiretroviral therapy (cART). Although efavirenz has been used in clinical practice for almost two decades, there is still a great need for deeper knowledge regarding the safety of efavirenz-containing treatment regimens [2]. The drug itself has several side effects, and presents a risk of even greater toxicity when co-administered with other drugs because of potential drug-drug interactions (DDI). Renal toxicity and impairments in hepatic function are among the most common cART-associated adverse effects [3, 4], and can be made more severe by pharmacokinetic DDI affecting the elimination rate of co-administered antiretrovirals and/or their accumulation in excretory organs [5].

ATP-binding cassette (ABC) and solute carrier (SLC) transporters are currently recognized as membrane proteins that profoundly affect the disposition of antiretroviral drugs, and are responsible for many clinically significant DDI [6]. Several members of the ABC efflux transporter superfamily are expressed in elimination organs and physiological barriers, and significantly affect the absorption, distribution and elimination of many different drugs [7, 8]. Notable ABC transporters of this kind include P-glycoprotein (*ABCB1*, MDR1), Breast Cancer Resistance Protein (*ABCG2*, BCRP) and Multidrug Resistance-Associated Protein 2 (*ABCC2*, MRP2). In the kidney and liver, these transporters mediate drug secretion from renal tubular cells and hepatocytes to the urine and bile, respectively. The organic cation transporters OCT1 (*SLC22A1*) and OCT2 (*SLC22A2*) are localized in the basolateral membranes of hepatocytes and proximal tubular cells, and mediate the uptake of drugs (primarily cationic ones) from the blood to the polarized cells [9]. The Multidrug and Toxin Extrusion Protein, MATE1 (*SLC47A1*), is localized in the canalicular membrane of hepatocytes and the apical membrane of proximal tubular cells, and mediates drug efflux to bile/urine, thereby enabling the final steps of drug excretion [10]. Inhibition of uptake or efflux transporters by a co-administered drug can thus cause DDI, resulting in sub-therapeutic or toxic plasma and tissue drug concentrations, and failure of therapy. Optimization of pharmacotherapies is therefore greatly facilitated by assessing the potential of novel drug entities and clinically used drugs to act as inducers, substrates, or inhibitors of membrane transporters and to cause DDI; this point is continuously stressed by the International Transporter Consortium and various medical regulatory agencies [11–13].

Efavirenz was confirmed to be neither a substrate nor an inducer of MDR1, but its inhibitory activity against MDR1 remains controversial [14–17]. It was shown to inhibit MRP transporters [18] and BCRP [19], albeit only at the relatively high concentration of 50 μM. There are also inconsistencies in reports of its inhibitory activity against OCT transporters: it failed to inhibit MPP^+^ uptake into HEK 239 cells stably transfected with human OCT1 or OCT2 [20], but a more recent study identified it as an inhibitor of OCT1-mediated tetraethylammonium uptake [21]. To the best of our knowledge, its inhibitory potential against MATE1 and MATE2-K has not yet been studied. Data on possible transporter-mediated DDI in efavirenz-containing cART are also lacking. Lamivudine, the NRTI most commonly co-administered with efavirenz, is known to be actively secreted to urine [22], with OCTs and BCRP being regarded as the most probable excretory transport proteins [23]. However, recent studies have suggested that the transcellular transport of lamivudine involves MATE1- and MATE2-K-mediated efflux rather than BCRP [24, 25], and that MATEs are the key transporters in the renal excretion of lamivudine and thus potential sites of DDI.

The aim of this study was to evaluate the inhibitory potency of efavirenz towards selected uptake (OCT1, OCT2) and efflux (MDR1, BCRP, MRP2, MATE1, MATE2-K) transporters. Building on *in vitro* uptake/accumulation assays that revealed significant inhibition of MATE1-mediated efflux as well as OCT1- and OCT2- mediated uptake of model substrates by efavirenz, we investigated possible OCTs/MATE-mediated DDI between efavirenz and lamivudine using transport assays across cellular monolayers and *in vivo* pharmacokinetics experiments in rats, focusing on lamivudine elimination and excretory organ disposition.

## Material and methods

### Chemicals

Radiolabeled metformin ([^14^C]-metformin, 49.3 mCi/mmol and 98mCi/mmol), lamivudine ([^3^H]-lamivudine, 5.2 Ci/mmol) and 1-methyl-4-phenylpyridinium ([^3^H]-MPP^+^, 80 Ci/mmol) were purchased from Moravek Biochemicals, California, USA. Minimum Essential Medium, Fetal Bovine Serum, HBSS buffer, HEPES, MES and ULTIMA scintillation cocktail were purchased from Sigma–Aldrich (St. Louis, Missouri, USA) or Invitrogen GmbH (Karlsruhe, Germany).

Efavirenz was obtained from the NIH AIDS Reagent Program. Gibco Opti-MEM reduced serum medium and bicinchoninic acid assay (BCA assay) kits were bought from Gibco (ThermoFisher Scientific). Pentobarbital (Nembutal) was purchased from Abbott Laboratories (Abbott Park, IL, USA). Other chemicals including transporter model inhibitors and fluorescent substrates were of analytical grade and obtained from Sigma–Aldrich.

### Cell cultures

The MDCKII parental cell line and MDCKII cells stably transduced for expression of the human transporters P-gp (MDCK-MDR1), BCRP (MDCK-ABCG2), or MRP2 (MDCK-MRP2) were provided by Dr. Alfred Schinkel (The Netherlands Cancer Institute, Amsterdam, The Netherlands). All the MDCK cell lines were cultured in DMEM medium, supplemented with 10% FBS. Singly-transfected MDCKII cell lines stably expressing human OCT1, OCT2, and MATE1 transporters, doubly-transfected MDCK-OCT1-MATE1 and MDCK-OCT2-MATE1 cells, and the vector control cell line MDCK-Co were prepared as described previously [26] and cultured in MEM medium supplemented with 10% FBS. All cells were routinely cultivated in antibiotic-free medium and periodically tested for mycoplasma contamination. Stable expression of all transporters was verified by qRT-PCR and uptake assays using appropriate fluorescence substrates. Cells from passages 10 to 25 were used in all *in vitro* studies. Parental human embryonic kidney 293 (HEK293)-cells were cultured, and HEK293-cells transiently transfected with MATE2-K were generated as previously described [24].

### Animals

Male Wistar rats were obtained from Biotest Ltd (Konarovice, Czech Republic) and maintained in 12/12-h day/night standard conditions with pellets and water *ad libitum*. Over-night fasted rats were anesthetized with pentobarbital (40 mg/kg) administered into the tail vein. All experiments were approved by the Ethical Committee of the Faculty of Pharmacy in Hradec Kralove (Charles University in Prague, Czech Republic) and performed in accordance with the Guide for the Care and Use of Laboratory Animals [27] and the European Convention for the protection of vertebrate animals used for experimental and other scientific purposes [28].

#### Accumulation of metformin

The singly-transfected OCT1, OCT2, MATE1 or control MDCK cells were seeded on 12-well plates at a density of 0.5 x 10^6^ cells per well and cultivated in standard cultivation medium MEM (Gibco) + 10% FBS. Forty-eight hours after seeding, uptake experiments with radiolabelled metformin were performed. Cells were washed with 500 μl pre-warmed HBSS buffer of pH 7.4 (for OCTs) or 8.0 (for MATE1). Solutions of [^14^C]-metformin (50 μM) in HBSS buffer of pH 7.4 or 8.0 with or without efavirenz were then added, and the resulting mixtures were left to stand for an accumulation period of 5 minutes. The incubation time was chosen based on previous studies indicating linearity of metformin uptake at this time point in the studied cell lines [24, 29]. After the accumulation period, the solutions were aspirated and the cells were washed three times with 500 μl ice-cold HBSS buffer of pH 7.4 or 8.0 before being lysed with 0.02% sodium dodecyl sulphate (SDS). The intracellular accumulation of radioactivity was detected using a liquid scintillation counter (Tri-Carb 2910 TR Perkin Elmer), and protein concentrations in the cell lysates were measured by the BCA protein assay. Net transporter-mediated uptake was obtained by subtracting the accumulation of metformin in vector control cells from that in drug transporter-overexpressing cells.

Experiments on the uptake of metformin in HEK 293-cells mediated by MATE2-K were performed as previously described [5]. The number of cells per well was 7.0x10^5^ for HEK-MATE2-K cells and 3.5x10^5^ for HEK-Co cells to compensate for differences in protein concentration in the two lines. To determine the inhibitory effect of efavirenz, this drug was added to the [^14^C]-metformin (50 μM) incubation medium at concentrations of 0.01 μM, 0.1 μM, 1 μM, 5 μM, 10 μM, 25 μM, or 50 μM. The reaction was stopped three minutes after efavirenz addition because previous experiments on the time dependence of metformin transport indicated that its uptake remains linear up to this point. The net uptake was then calculated as described above.

#### Accumulation experiments in MDCK-MDR1, MDCK-BCRP, and MDCK-MRP2 cells

ABC transporter-expressing MDCK cells were seeded in 96-well plates at a density of 25 x 10^3^ cells per well and cultivated in DMEM medium supplemented with 10% FBS. Forty-eight hours after seeding, the cells were used in accumulation experiments with Hoechst 33342 (a common fluorescent substrate of MDR1 and BCRP) or efflux assays with Calcein (a fluorescent substrate of MRP2). Briefly, at the end of the 48 hours, the medium was aspirated and the cells were washed with pre-warmed PBS and then preincubated with efavirenz in Opti-MEM for 30 minutes. In the Hoechst 33342 assays, a solution of Hoechst 33342 (50 μg/ml) in Opti-MEM was added and allowed to accumulate for 30 minutes. The experiment was stopped by aspirating the medium and washing the cells twice with ice cold PBS. Intracellular fluorescence was then measured using excitation and emission wavelengths of 350 nm and 465 nm, respectively. In the calcein efflux assays, a solution of calcein AM in Opti-MEM with or without efavirenz was added after the pre-incubation phase and allowed to accumulate for 15 minutes. The solutions were then aspirated, the cells were washed with pre-warmed PBS, Opti-MEM with or without inhibitors was added, and the resulting solutions were left to stand for 60 minutes. Finally, the efflux phase was terminated by aspirating the solutions and washing the cells twice with ice cold PBS. Calcein fluorescence was measured using excitation and emission wavelengths of 496 nm and 516 nm, respectively.

### Transcellular transport assays

Transport experiments employing polarized monolayers of MDCK-OCT1, MDCK-OCT2, or MDCK-MATE1 cell lines as well as doubly transfected MDCK-OCT1-MATE1 or MDCK-OCT2-MATE1 lines and the non-transfected parental control MDCK-Co cells grown on Transwell 3402 cell culture inserts (3.0 μm pore size, 24 mm diameter; Costar, Corning, NY) were performed as described previously [24–26]. The number of cells per well was 0.5 x 10^6^ in all experiments, and the cells were incubated for 3 days to confluence in standard cultivation medium MEM (Gibco) + 10% FBS. After 3 days’ cultivation, medium was removed from both sides and the cells of the monolayer were washed using pre-warmed PBS. Experiments were initiated by adding HBSS buffer containing 10 mM HEPES (pH 7.4) or 10 mM MES (pH 6.0) with or without [^3^H]-lamivudine (10 nM) or [^3^H]-MPP^+^ (2 nM) to either the apical or basolateral compartment. The pH of the medium on the basolateral side was 7.4, while that on the apical side was set to 6.0 (adjusted by adding HCl or NaOH, when necessary) to ensure optimal MATE1 transport activity. Cells were incubated at 37°C in 5% CO_2_ for 2 hours. At the end of the incubation period, 50 μl aliquots were sampled and the rest of the medium was immediately removed. The cells were then washed twice with ice-cold PBS, the membranes supporting cellular monolayers were excised, and the cells were dissolved in a 0.02% SDS solution. The radioactivity of the collected samples and lysed monolayers was measured by liquid scintillation counting (Tri-Carb 2910 TR Perkin Elmer), and the protein concentration of the cell lysates was quantified using the BCA protein assay. Transport rates were recorded in pmol per mg protein in the cellular monolayer.

### In vivo pharmacokinetic experiments

Male Wistar rats (260–320 g) were anesthetized and the *vena jugularis*, bile duct and urinary bladder were cannulated for sample collection. Efavirenz (10 μM) or cimetidine (300 μM) were applied in physiological saline containing 1% DMSO into the *vena saphena* at a volume of 4 μl/ 5 g of animal body weight, giving doses 2.53 mg/kg and 60.6 mg/kg animal weight, respectively. The dose of efavirenz was chosen to achieve a drug concentration in the plasma corresponding to that seen in humans (0.4–48 μM; median 6.9 μM) [30]. Cimetidine was used at a dose producing plasma concentrations of about 104 μM (calculated based on its distribution volume in rats [31]), corresponding to the concentration expected to inhibit MATE and OCT transporters [32]. After 20 minutes, solutions of [^3^H]-lamivudine (10 μCi/ml; 1.59 μM) containing the appropriate inhibitor (or an equivalent volume of solvent in control animals) were administered at a volume of 4 μl per 5 g of rat body weight. This concentration was the lowest sufficient to ensure sensitive scintillation analysis of all the blood, bile and urine samples, which were collected for 240 minutes after the administration of lamivudine. The radioactivity of the collected samples was measured by liquid scintillation counting (Tri-Carb 2910 TR Perkin Elmer). At the end of the experiment, the animal was euthanized and its kidney and liver tissue were excised to determine their lamivudine retention. Three distinct parts of the kidney and liver were sampled, weighed (50–100 mg per piece), and dissolved in tissue solubilizer (Solvable; PerkinElmer Life and Analytical Sciences) according to the manufacturer´s protocol. The tissue lysates were then analysed for radioactivity and the amount of [^3^H]-lamivudine in the tissue was calculated.

### Data analysis

Statistical analyses were performed using GraphPad Prism, version 7.03 (GraphPad Software, San Diego, CA). *P* values were calculated using multiple two-tailed *t*-tests or one-way ANOVA followed by Dunnett’s or Bonferroni’s multiple comparison test, as appropriate. A *p* value of ≤ 0.05 was considered statistically significant. The half-maximal inhibitory concentration (IC_50_) was calculated by non-linear regression analysis using sigmoidal Hill kinetics. Plasma concentrations obtained from *in vivo* experiments were fitted to a two-compartment model using the SAAM II (v2.3) software package to obtain AUC (area under curve) and β (hybrid constant of the elimination phase) values. Subsequently, total body clearance was calculated as dose/AUC_0–∞_ and elimination half-life was calculated as 0.693/β.

## Results

### Inhibition of metformin uptake by efavirenz in MDCKII cells overexpressing OCT1, OCT2, and MATE1, and in HEK293 cells overexpressing MATE2-K

**Fig 1:**
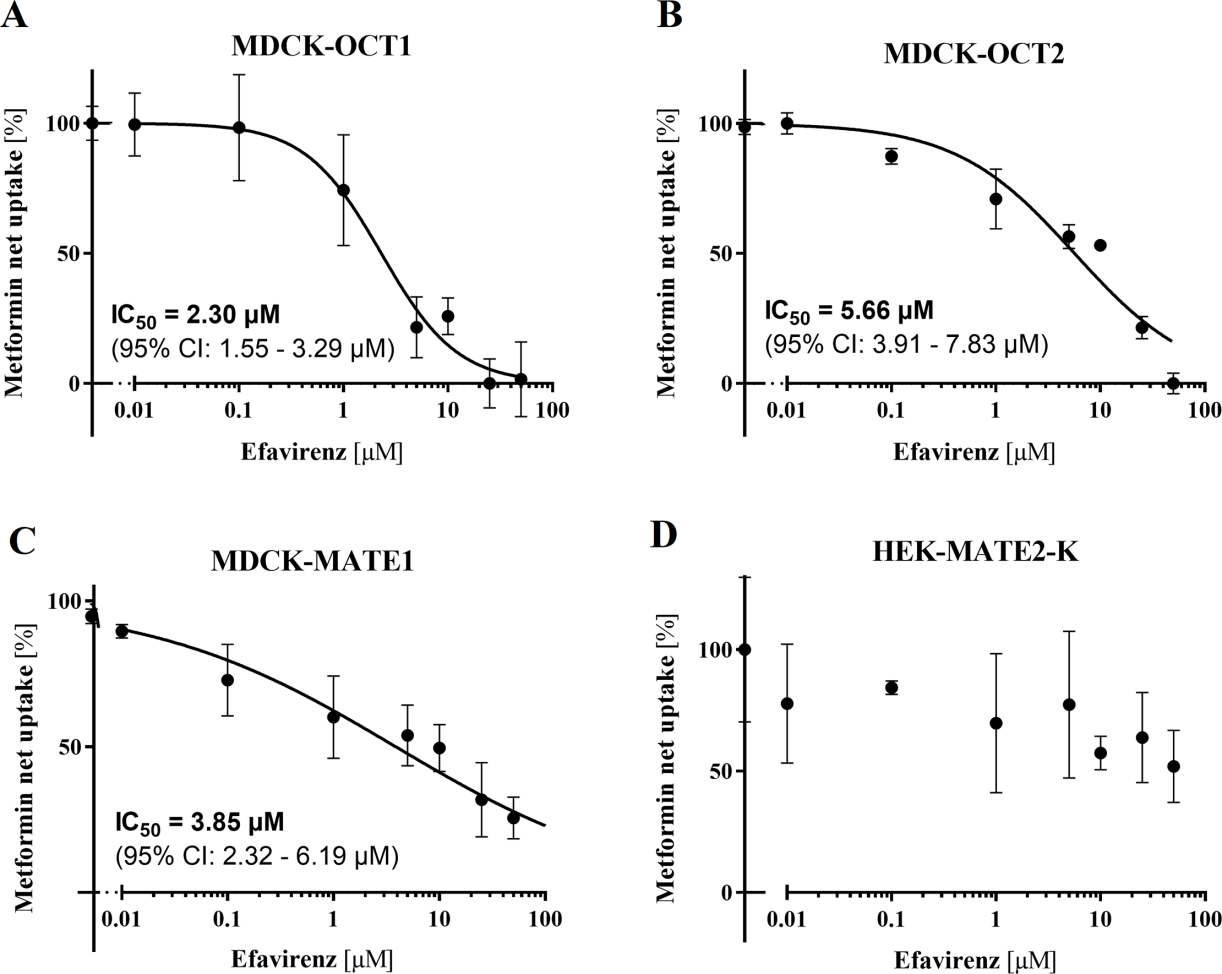
**Inhibition of OCT1-, OCT2-, MATE1-, and MATE2-K-mediated net metformin uptake by efavirenz (0.01–50** μ**M) in MDCK-OCT1 (A), MDCK-OCT2 (B), MDCK-MATE1 (C), and HEK-MATE2-K (D) cells.** Data are shown as means ± SD of at least four experiments performed in triplicate (MDCK cell lines) or three experiments performed in duplicate (HEK-MATE2-K cells). Calculated IC_50_ values and the associated 95% confidence intervals are shown where appropriate.

Uptake assays with radiolabelled metformin, a common substrate of OCT and MATE transporters [33], were performed to evaluate and quantify the inhibitory effect of efavirenz on MATE1, OCT1, and OCT2. In the presence of efavirenz, we observed significant inhibition of net transporter-mediated [^14^C]-metformin uptake into MDCK-OCT1, MDCK-OCT2, and MDCK-MATE1 cells, with IC_50_ values of 2.30 μM, 5.66 μM and 3.85 μM, respectively (Fig 1A, 1B and 1C). These findings indicate that efavirenz inhibits the OCT1, OCT2, and MATE1 transporters *in vitro*. To determine whether it also inhibits transport mediated by MATE2-K, further metformin uptake assays were performed using HEK293-cells transiently overexpressing this transporter (Fig 1D). At the highest tested concentration (50 μM), efavirenz inhibited MATE2-K-mediated metformin transport by 48.1% (*p* = 0.119, one-way ANOVA followed by Dunnett's multiple comparisons test).

### Inhibition of transcellular transport and intracellular accumulation of MPP+ and lamivudine in the presence of efavirenz

**Fig 2:**
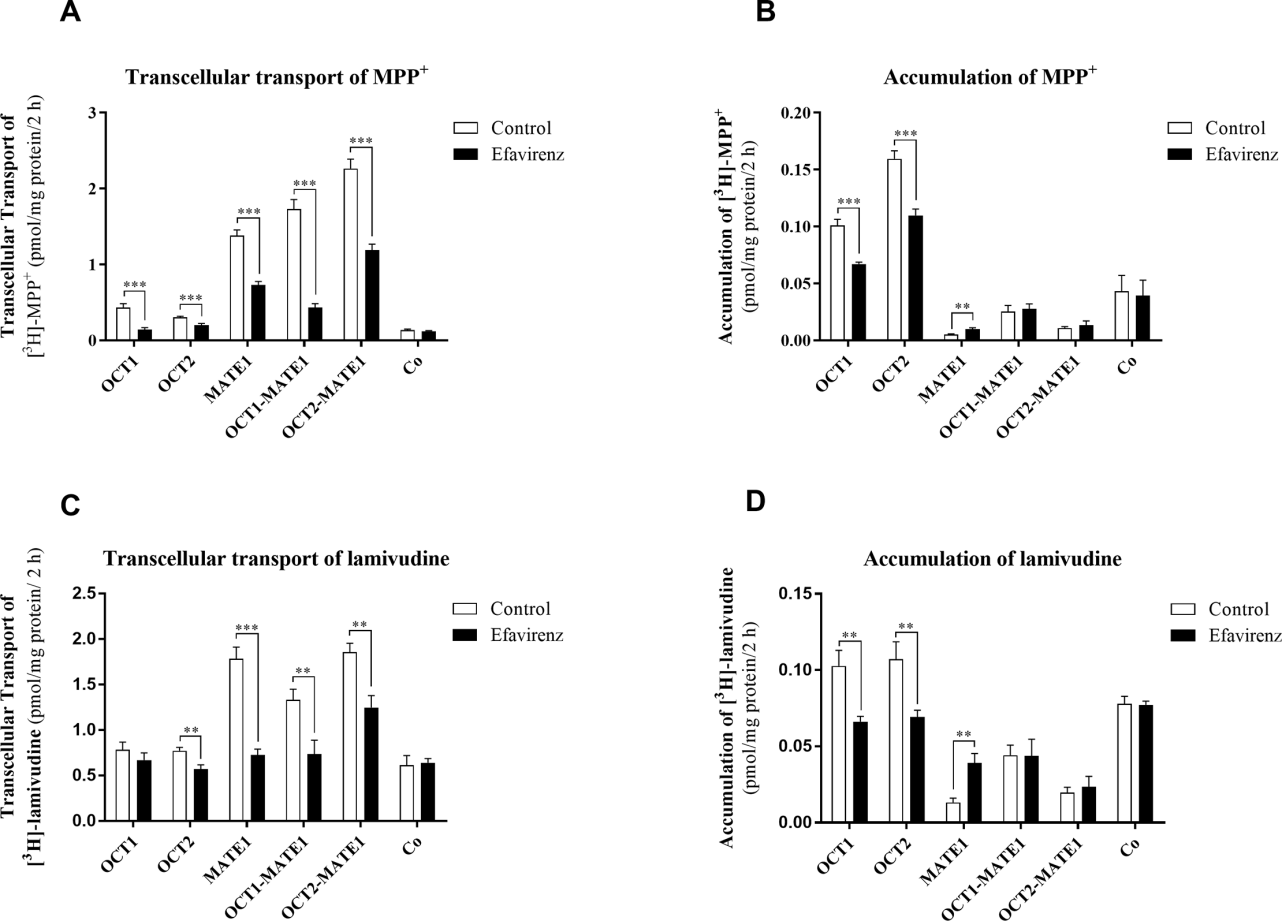
**Effect of efavirenz (10** μ**M) on transcellular transport of 2 nM[**^**3**^**H]-MPP**^**+**^ **(A) and 10 nM [**^**3**^**H]-lamivudine (C) from the basal to the apical compartment, and intracellular accumulation of [**^**3**^**H]-MPP+ (B) and [3H]-lamivudine (D) in monolayers of MDCK-OCT1, MDCK-OCT2, MDCK-MATE1, MDCK-OCT1-MATE1, MDCK-OCT2-MATE1, and control MDCK-Co cells over 2 hours.** Data are shown as means ± SD of at least three independent experiments performed in duplicate. Results were analysed using the multiple t-test **P≤ 0.01 ***P ≤ 0.001.

We performed follow-up transport experiments to evaluate the effects of efavirenz on vectorial OCTs/MATE1-mediated transcellular transport of MPP^+^, another model substrate of these transporters [26]. Inhibition of basolateral-to-apical transport of [^3^H]-MPP^+^ (2 nM) by efavirenz (10 μM) was observed in monolayers of MDCK-OCT1, MDCK-OCT2, and MDCK-MATE1 cells, as well as in doubly transfected MDCK-OCT1-MATE1 and MDCK–OCT2-MATE1 cells (Fig 2A), while no inhibitory effect of efavirenz was detected in control MDCK-Co cells (Fig 2A). In the monolayers of singly-transfected OCT1- and OCT2- cells, efavirenz significantly reduced the intracellular accumulation of [^3^H]-MPP^+^. Conversely, efavirenz-induced inhibition of MATE1-mediated efflux on the apical membrane of polarized monolayers of MDCK-MATE1 cells increased [^3^H]-MPP^+^ accumulation. The intracellular accumulation of [^3^H]-MPP^+^ was unaffected by efavirenz in doubly-transfected MDCK-OCT1-MATE1 and MDCK-OCT2-MATE1 cells, presumably reflecting simultaneous inhibition of both OCT-mediated uptake and MATE1-mediated efflux mechanisms (Fig 2B).

Similar results were observed in experiments using lamivudine, a substrate of OCT1, OCT2, and MATE1 [23, 24]. Efavirenz (10 μM) significantly inhibited transcellular transport of [^3^H]-lamivudine (10 nM) across monolayers of singly-transfected MDCK-OCT2 and MDCK-OCT1 cells as well as doubly-transfected MDCK-OCT1-MATE1 and MDCK-OCT2-MATE1 lines, but not in control MDCK-Co cells (Fig 2C). In the presence of 10 μM efavirenz, intracellular accumulation of [^3^H]-lamivudine (10 nM) was significantly reduced in MDCK-OCT1 and MDCK-OCT2 cells and enhanced in MDCK-MATE1 cells, but remained unaffected in doubly-transfected OCT1-MATE1 and OCT2-MATE1 cells (Fig 2D). These transport experiments confirm that efavirenz is an *in vitro* inhibitor of OCT1, OCT2, and MATE1, and reveal a potential transporter-mediated drug-drug interaction between efavirenz and lamivudine.

### Inhibitory effects of efavirenz on MDR1, BCRP and MRP2 transporters

**Fig 3:**
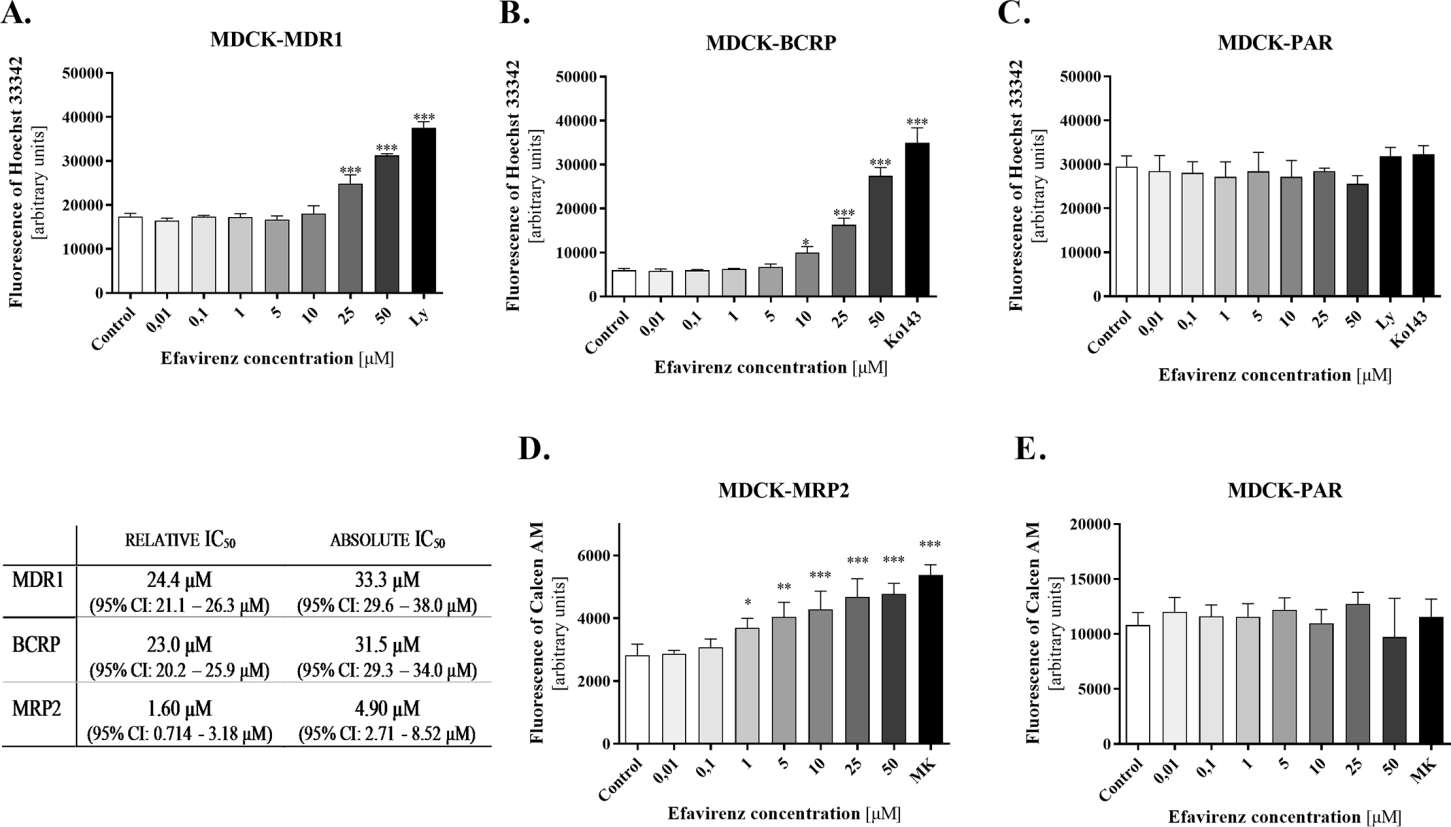
Inhibitory effect of efavirenz on MDR1, BCRP, and MRP2 transporters. Efavirenz was applied at concentrations of 0.01–50 μM to evaluate its inhibition of Hoechst 33342 accumulation in the MDCK-MDR1 (A), MDCK-BCRP (B), and parental MDCK (C) cell lines, as well as its inhibition of calcein efflux from the MDCK-MRP2 (D) and parental MDCK (E) lines. Model inhibitors of MDR1, BCRP and MRP2, namely LY335979 (LY, 1 μM), Ko143 (2.5 μM), and MK571 (10 μM), respectively, were used as positive controls at the minimal concentrations causing maximal inhibition in the corresponding cellular system and assay. Relative and absolute IC_50_ values were calculated as the concentrations of efavirenz needed to achieve 50% transporter inhibition. The relative IC_50_ was determined from the maximum and minimum extremes of the relevant non-linear regression plot showing the inhibitory effect of efavirenz alone (maximal inhibition = 100%, non-inhibited control = 0%). The absolute IC50 was determined from the relevant non-linear regression plot of the inhibitory effect of the control inhibitor (100%) and the accumulation observed in control non-inhibited cells (0%). Data were analyzed by one-way ANOVA followed by Bonferroni’s multiple comparison test (* P ≤ 0.05, ** P ≤ 0.01, *** P ≤ 0.001) and are presented as means ± SD of at least three experiments (n = 3–5) performed in triplicate.

The Hoechst 33342 accumulation assays revealed an inhibitory effect of efavirenz on MDR1 and BCRP when applied at concentrations ≥ 25 μM and 10 μM, respectively (Fig 3). However, its inhibitory potency was lower than that of the control inhibitors LY335979 and Ko143. This could be due to incomplete inhibition, but may also be a consequence of the limited range of efavirenz concentrations tested in the study; higher concentrations could not be used because of the drug’s cytotoxicity in all the accumulation assays and MDCK cell lines at the highest tested concentration (100 μM). Therefore, both relative and absolute IC_50_ values are reported (Fig 3).

In efflux assays using calcein AM (0.25 μM), efavirenz inhibited the efflux of fluorescent calcein from MDCK-MRP2 cells but not from the parental MDCK cell line (Fig 3). Efavirenz thus seemingly inhibits the MRP2 drug transporter at concentrations ≥ 1 μM. As in MDR1- and BCRP-expressing cells, the level of inhibition observed at an efavirenz concentration of 50 μM was lower than that induced by the control inhibitor when applied at the minimum concentration required for maximal inhibition (Fig 3).

### Effect of efavirenz on renal excretion and kidney accumulation of lamivudine in vivo

To better assess the relevance of the Oct- and Mate1-mediated DDI between efavirenz and lamivudine as observed *in vitro* in cellular monolayers, we performed an *in vivo* pharmacokinetic study in Wistar rats. Cimetidine was used as a model inhibitor of Oct and Mate transporters [34].

**Fig 4:**
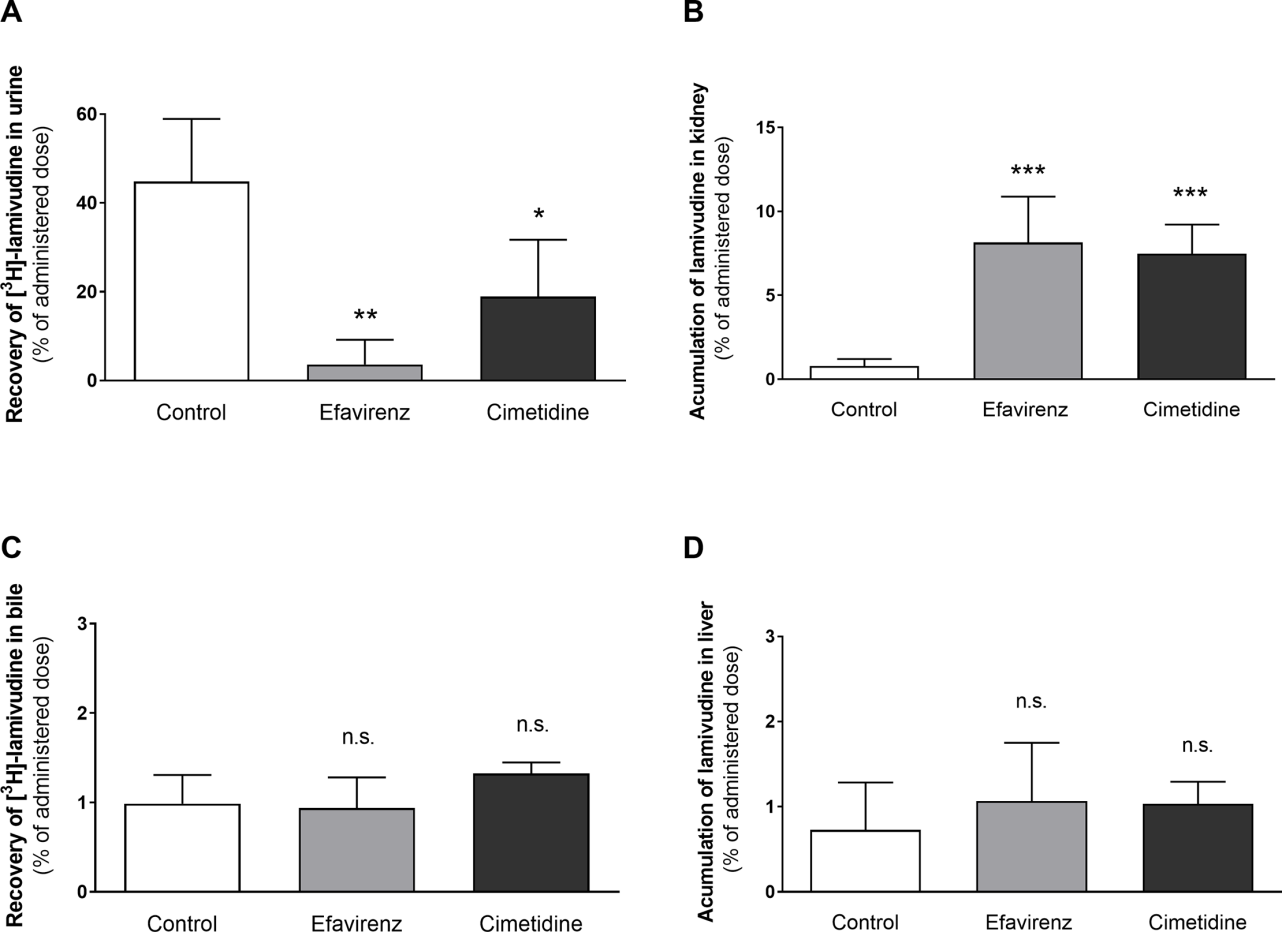
**Total recovery of [**^**3**^**H]-lamivudine from urine (A) and bile (C), and its accumulation in renal (B) and liver (D) tissue at 240 minutes after i.v. administration with or without co-administration of efavirenz (2.53 mg/kg) or cimetidine (a control inhibitor of the OCT and MATE transporters; 60,6 mg/kg).** Data are shown as means ± SD (n = 5) and were analysed using one-way ANOVA followed by Dunnett`s multiple comparison test * P ≤ 0.05, *** P ≤ 0.001, n.s. not significant.

At 240 min after treatment, 44.9% of the intravenously administered lamivudine was recovered from the rat urine, and only 0.976% was excreted into the bile. Co-administration of efavirenz significantly reduced (to 8.1%) the urinal recovery of [^3^H]-lamivudine (Fig 4A), while co-administration of cimetidine reduced it to 42.1%. The recovery of [^3^H]-lamivudine from bile was not significantly changed by co-administration of either efavirenz or cimetidine, reaching 95.0% and 134.4% of the control value, respectively (Fig 4C).

Efavirenz significantly increased the accumulation of lamivudine in the kidney tissue (by a factor of 10.3) during the first 240 minutes after administration, probably by preferentially inhibiting lamivudine efflux from the renal cells (Fig 4B). The presence of cimetidine increased the accumulation of lamivudine in the kidney 9.84-fold. No effect of efavirenz or cimetidine on tissue accumulation was observed in the liver (Fig 4D).

### Effect of efavirenz on lamivudine AUC (plasma levels) in vivo

Co-administration of efavirenz significantly reduced clearance of lamivudine (by 41.4%), prolonged its elimination half-life (by 189%) and thus increased lamivudine AUC_0-∞_ (by 129%). The observed effect of efavirenz on the pharmacokinetics of lamivudine was similar to that of the model inhibitor cimetidine, which reduced the total body clearance of lamivudine by 38.9% and increased its AUC_0-∞_ by 70.6% (see Table 1).

**Table 1:** Effects of efavirenz and cimetidine on the pharmacokinetics of lamivudine in rats.

	*AUC_0-∞_* *[pg x min/ml]*	*CL* *[ml/min]*	*t_1/2_* *[min]*
*Control*	27080 ± 8137	3.266 ± 1.055	148.7 ± 62.80
+ *efavirenz*	62130 ± 23670*	1.908 ± 0.920*	430.4 ± 237.8**
+ *cimetidine*	46210 ± 13180*	1.960 ± 0.467*	271.9 ± 119.0

Data are presented as means ± SD (n = 5).

*P ≤ 0.05

**P ≤ 0.01 compared with the control group

## Discussion

The results presented here show for the first time that efavirenz is a potent inhibitor of MATE1, OCT1, OCT2, and MRP2 capable of causing transporter-mediated DDI with lamivudine *in vitro* and *in vivo*.

Although Jung et al. [20] did not detect any inhibitory effect of efavirenz (at a concentration of 5 μM) on OCT1 or OCT2 when using MPP^+^ as a substrate, a subsequent study by Moss et al [21] identified efavirenz as an OCT1 inhibitor because OCT1-overexpressing KCL2 cells were observed to exhibit a 50.0% reduction in tetraethylammonium accumulation upon treatment with 50 μM efavirenz [21]. Our *in vitro* data for MDCK cells transfected with OCT1, OCT2, or MATE1 show that efavirenz efficiently inhibits all these transporters and affects the cellular handling of two important model substrates, MPP^+^ and metformin; its calculated IC_50_ values for the inhibition of metformin uptake by OCT1, OCT2, and MATE1 are 2.30, 5.66 and 3.85 μM, respectively. The lack of interactions observed by Jung et al. may be due to aspects of their study design such as the cellular models that were employed, the low concentration of efavirenz that was tested (5μM), and/or the relatively short (1 min) incubation times. Additionally, the adherence of efavirenz to some plastic surfaces [17] may have contributed to the variation in the experimental results reported by different research teams, potentially causing false negative results or artificially increasing the IC_50_ values obtained in inhibitory assays.

To the best of our knowledge, this is the first study to identify efavirenz as a potent inhibitor of the MATE1 transporter and to quantify its inhibitory activity against OCT uptake proteins. Based on the tissue locations of these transporters, efavirenz could plausibly cause DDI affecting the renal or hepatic elimination of co-administered drugs.

Previous studies showed that lamivudine is a high affinity substrate of MATE1 and MATE2-K [24, 25], and suggested these transporters play key roles in the renal tubular secretion of lamivudine, which is the main pathway of lamivudine elimination [35–37]. Here we hypothesized that the co-administration of efavirenz with lamivudine could cause DDI at the level of renal elimination. We first demonstrated significant *in vitro* inhibition of lamivudine transcellular transport across all MATE1-overexpressing and MDCK-OCT2 monolayers by efavirenz and subsequently performed *in vivo* pharmacokinetic experiments in rats. The *in vivo* study was performed because current recommendations state that evaluations of inhibitory drugs should be performed using concentrations relevant for the location of the transporter [38, 39]. Specifically, this means that the K_i_ (inhibition constant) or IC_50_ observed *in vitro* should be at least 50 times the unbound c_max_ (peak plasma concentration) to justify *in vivo* studies on renal uptake and efflux transporters and hepatic efflux transporters using inhibitors identified *in vitro*. The average plasma concentration (c_p_) of efavirenz observed therapeutically in responding HIV-positive patients is ~8.7 μM [40], and its median unbound percentage in humans is 0.63% because it binds extensively to plasma proteins [41]. Consequently, 50x the unbound concentration of efavirenz in elimination organs is around 2.7 μM, which lies within the 95% CI of the IC_50_ values determined for OCT1 and MATE1 (Fig 1).

The observed elimination profile of lamivudine in Wistar rats is broadly consistent with its pharmacokinetics in humans: around 70% of the administered dose was recovered from the urine unchanged [22]. Efavirenz significantly reduced the total clearance of lamivudine and its recovery in the urine (by 91.9%) while enhancing its accumulation in kidney stroma 10.3-fold (Fig 4A and 4B). Similarly, the clearance of lamivudine was reduced and AUC_0-∞_ increased substantially when co-administered with efavirenz or cimetidine (Table 1). This effect differs from that of the OCTs/MATEs inhibitor trimethoprim, which reduced the renal clearance of lamivudine by half in rats and also raised the plasma concentration of lamivudine but did not impair its accumulation in the renal cortex [36]. This interaction pattern was suggested to result from inhibition of both OCT2-mediated uptake at the basolateral membrane and apical membrane-localized efflux mediated by MATE1. The interaction profile of efavirenz observed in the current study indicates a more profound inhibition of efflux mechanisms to the urine, leading to enhanced accumulation of lamivudine in renal tissue, which was not observed with trimethoprim [36]. This difference might be explained by different relative expression of OCT 1/2 and MATE1 in the rat kidney compared to the human transporters expression in the MDCK cellular models. The control inhibitor cimetidine exhibited the same inhibitory pattern as efavirenz, in keeping with previously published *in vivo* studies [32, 36], suggesting that competitive inhibition of MATEs rather than OCT2 is the process responsible for the renal DDI caused by cimetidine [32, 33]. The dose of cimetidine used in this work was higher than that used previously because we wanted to achieve a plasma concentration exceeding the previously published IC_50_ for hOCT2 and rOCT2 [24, 32]. Despite the use of this high concentration, the inhibitory pattern observed for cimetidine in our *in vivo* studies still seems to reflect predominant inhibition of MATE transporters rather than renal OCT2.

Based on our in vivo observations we suggest that, like the DDI between cimetidine and lamivudine, the DDI between efavirenz and lamivudine probably results from inhibition of the apically localized efflux component rather than inhibition of basolateral membrane-localized influx. It is reasonable to assume that there may be some other efflux protein in addition to MATE1 (most probably an ABC protein such as MDR1, BCRP or MRP2) that contributes to this DDI. Nevertheless, despite some reports suggesting that lamivudine is a substrate of BCRP [42] and MDR1 [43], our recent study provided no evidence that it is a substrate of MDR1, BCRP, or MRP2 in MDCK cells *in vitro* or *in situ* in dually perfused rat term placentas [25]. Moreover, efavirenz shows only weak inhibitory activity against ABC transporters, with absolute IC_50_ values greatly exceeding its therapeutically achieved plasma concentrations. Consequently, the inhibition of MDR1- and BCRP-mediated lamivudine transport by efavirenz is very unlikely to be relevant *in vivo*.

Efavirenz has also been described as a potent inhibitor of MRP transporters [18]; in this work, its IC_50_ value against MRP2 was found to be comparable to its plasma concentrations. Therefore, MRP2 can be regarded as another potential site of transporter-mediated DDI involving efavirenz. Since lamivudine is not a substrate of MRP2 [25], we do not believe that its increased accumulation in renal tissue is attributable to MRP2 inhibition by efavirenz. Nevertheless, it may be necessary to consider potential MRP2-mediated DDI when combining efavirenz with drugs that are MRP2 substrates.

The *in vivo* pharmacokinetics data from our study on rats revealed that efavirenz inhibits renal excretion of lamivudine and strongly increases its accumulation in kidney tissue. Interestingly, recent clinical data show that co-administration of lamivudine (150 mg twice daily) with efavirenz (600 mg once daily) increased the C_min_ of lamivudine by 265% but had no effect on its C_max_ or AUC [44]. The authors ascribed the changes in C_min_ to DDI affecting drug-metabolizing enzymes. However, based on the results presented herein, we hypothesize that the clinically observed increase in lamivudine C_min_ might result from decreased renal clearance of lamivudine caused mainly by MATE1-mediated interactions. Lamivudine is generally considered to be a well-tolerated antiretroviral with a low incidence of side effects [2]. However, several cases of nephrotoxicity with renal tubular acidosis resulting from treatments involving lamivudine have been reported [45, 46]. It therefore seems plausible that the efavirenz-induced increase of lamivudine retention in renal tissue could cause a risk of enhanced nephrotoxicity, particularly in patients with a chronic kidney disease, the major comorbidity in HIV-infected patients [47]. This safety issue should also be taken into consideration when efavirenz is combined with other antiretrovirals such as emtricitabine, which was recently identified as another substrate of MATE1 [48].

To conclude, this study provides the first evidence of significant inhibition of MATE1, OCT1, OCT2, and MRP2 transporters by efavirenz, and of OCT/Oct and MATE1/Mate1-mediated interactions between efavirenz and lamivudine leading to reduced lamivudine clearance to urine, prolonged elimination half-life and increased retention of this drug in renal tissue. If confirmed in clinical settings, this DDI should be considered when administering this drug combination to HIV patients with elevated risks of nephrotoxicity.

## Abbreviations

ABCB1: P-glycoprotein
ABCG2: breast cancer resistance protein
cART: combination antiretroviral therapy
ANOVA: one-way analysis of variance
DMEM: Dulbecco’s modified eagle medium
DMSO: dimethyl sulfoxide
MDCK: Madine-Darby canine kidney cells
MATE: Multidrug and Toxin Extrusion Protein
NRTI: nucleoside reverse transcriptase inhibitors
OCT: organic cation transporter
